# Examination of post-exercise microvascular reactivity in healthy adults

**DOI:** 10.1007/s00421-025-05906-y

**Published:** 2025-07-22

**Authors:** Rian Q. Landers-Ramos, Thomas Silva, Devon A. Dobrosielski, Nicolas D. Knuth

**Affiliations:** Department of Kinesiology, Towson University, Towson, MD 21252 USA

**Keywords:** Microvascular reactivity, NIRS, Blood volume, Acute exercise

## Abstract

**Aim:**

Skeletal muscle blood volume responds to the metabolic demands of exercise and augmented microvasculature reactivity. We sought to explore the effects of exercise intensity (maximal vs. submaximal) on near-infrared spectroscopy (NIRS)-derived blood volume and microvascular reactivity in the acute post-exercise timeframe.

**Methods:**

Healthy individuals (*N* = 18) between 18 and 35 years completed a vascular occlusion test (VOT) followed by a maximal cycling test. A second VOT was performed 15-min post-exercise. One week later, the protocol was repeated before and after a submaximal bout of cycling (60% VO_2_ peak). NIRS was used to assess total hemoglobin (tHb) (i.e., blood volume) before, during, and after exercise, as well as muscle oxygen consumption (mVO_2_) and microvascular reactivity (StO_2_% s^−1^) pre- and post-exercise.

**Results:**

Compared with pre-exercise, tHb was elevated at the end of exercise (*p* < 0.001) and remained elevated 15-min post-exercise (*p* < 0.001) regardless of trial (combined means pre: 5.39 ± 0.82, during: 14.01 ± 1.73, and post-exercise: 10.89 ± 1.24 O.D.). mVO_2_ was greater post-exercise in the max vs. submax trial (− 0.36 ± 0.12 vs. − 0.22 ± 0.11% s^−1^; *p* < 0.001). Compared with pre-exercise, microvascular reactivity was unchanged following the max trial (1.91 ± 0.61 vs. 1.71 ± 0.61% s^−1^; *p* = 0.079) but was greater following the submax trial (1.72 ± 0.43 vs. 1.98 ± 0.59; *p* = 0.007).

**Conclusion:**

Cycling at a submaximal, but not maximal, intensity results in augmented post-exercise microvascular reactivity, while post-exercise increases in skeletal muscle blood volume were found regardless of exercise intensity.

## Introduction

Skeletal muscle blood flow increases during exercise in proportion to the metabolic demand of the active tissue (Andersen and Saltin 1985; Bangsbo and Hellsten 1998; Tschakovsky et al. 2002; Sarelius and Pohl 2010) and remains elevated between 10 and 90 min after exercise (Halliwill et al. 1996; Moynes et al. 2013) to aid recovery and the return to homeostasis (Thijssen et al. 2006). This can be observed through localized changes in muscle blood volume in healthy adults (Newman et al. 1997; Higaki et al. 2021). The extent to which post-exercise blood flow remains elevated is thought to be intensity-dependent, with greater intramuscular blood flow or blood volume in the acute recovery timeframe following heavier exercise (Newman et al. 1997; Tschakovsky et al. 2002; Joyner and Casey 2015). However, most studies have observed this with exercise recruiting small muscle groups (i.e., handgrip or knee extension) (Andersen and Saltin 1985; Rådegran and Saltin 1999; Tschakovsky et al. 2002; Moynes et al. 2013; Joyner and Casey 2015; Larsen et al. 2015, 2019; Izumi et al. 2024). In contrast, a few studies have investigated blood flow or intramuscular blood volume changes following dynamic exercise that recruits larger muscle mass (Rowell 1997). Intensity-dependent differences in blood flow to a particular region of muscle may not be evident with greater muscle mass exercise, such as cycling, as the demands for blood are shared among a larger quantity of recruited muscle. This presents a significant gap in our translational understanding of recovery for sport and rehabilitation, as athletic movements typically involve dynamic, multi-joint movements recruiting large amounts of muscle mass.

Increased blood flow to exercising skeletal muscle is made possible, in part, through vasodilation of the large (i.e., macrovascular) and small (i.e., microvascular) blood vessels (Joyner and Casey 2015). However, high exercise intensity may impair vasodilatory function in the acute post-exercise timeframe, hindering recovery from intense exercise. At the macrovascular level, high-intensity exercise (> 80% VO_2_ max) typically results in a decrease in flow-mediated dilation (FMD) when measures are taken less than 30 min after exercise (Goto et al. 2003; Birk et al. 2012; Johnson et al. 2012b; Dawson et al. 2013). In contrast, low–moderate-intensity exercise reportedly causes an increase in post-exercise FMD (Harvey et al. 2005; Padilla et al. 2006; Harris et al. 2008; Zhu et al. 2010; Johnson et al. 2012a). At rest, FMD is correlated with near-infrared spectroscopy (NIRS)-based measures of skeletal muscle microvascular reactivity (McLay et al. 2016a; Soares et al. 2019), suggesting that post-exercise responses in the skeletal muscle microvasculature may also mirror these macrovascular responses (Robinson et al. 2018; Caldwell et al. 2023). However, microvascular responses following different intensities of exercise have not been adequately examined. For example, increases in microvascular reactivity have been documented following submaximal plantarflexion exercise (Meneses et al. 2018) and following moderate continuous exercise but not high-intensity interval exercise (Sweet et al. 2024). On the other hand, microvascular reactivity has reportedly been reduced following both moderate-intensity aerobic exercise (Caldwell et al. 2023) and a standard exercise stress test (i.e., high-intensity), even in healthy adults (Nardone et al. 2021). These conflicting findings warrant more research to better understand the impact of exercise intensity on post-exercise microvascular reactivity.

The acute recovery period following exercise can serve as a transient physiological timeframe that may predict future clinical outcomes even in healthy individuals (Luttrell and Halliwill 2015). A better understanding of the intensity-dependent microvascular responses to large muscle mass exercise in the acute post-exercise timeframe can also help to inform muscular recovery practices in sport and rehabilitation. Accordingly, the purpose of this study is to examine NIRS-derived blood volume and microvascular reactivity responses to maximal and submaximal cycling exercise. We hypothesize that NIRS-derived blood volume in the vastus lateralis (VL) will be increased relative to baseline both during and after cycling exercise, with similar changes in the maximal and submaximal trials. Further, we hypothesized that NIRS-derived microvascular reactivity in the VL would be attenuated 15 min following maximal cycling but would be greater following submaximal cycling.

## Methods

### Participants

This study was approved by the Towson University Institutional Review Board (IRB #2036). Written informed consent was obtained from all participants and all study procedures adhered to those outlined in the Declaration of Helsinki. All participants provided consent to publish. Participants between the ages of 18–35 years were recruited from the Baltimore, Maryland region. Male and female participants were recruited, and information about health history, medication use, and date of the menstrual cycle (first date of the last menstrual cycle in female participants) was documented through self-report. All participants were recreationally active defined as participation in ≥ 30 min/day of moderate-intensity cardiovascular and/or resistance training on 3–5 days/week (Garber et al. 2011). Physical activity was measured via self-report. Participants were asked to quantify the approximate number of minutes per week they spent performing resistance and aerobic exercise days in the previous week. Inactive (< 20 min of structured exercise/day on > 2 days/week) and trained or competitive athletes were excluded from this study due to the potential contribution of training status to our study outcomes. Participants were excluded if they had a body mass index (BMI) > 30 kg/m^2^, were regular smokers (within the past 6 months), had musculoskeletal or other injuries that would prevent them from completing a high-intensity exercise bout, and if they had previously diagnosed coronary heart disease or congenital heart disease, or any health condition in which high-intensity exercise is contraindicated. Participants were also excluded if they were currently taking prescription-strength anti-inflammatory medications or over-the-counter antioxidants on a regular basis.

### Study design

This study involved two visits to our labs, separated by 1 week. Visit 1 included baseline assessments (height, weight, blood pressure, and body composition) performed in the morning between 6:00 and 11:00 AM. This was followed by a pre-exercise vascular occlusion test (VOT). Participants then performed a maximal exercise trial during which intramuscular blood volume was measured in the final 30 s, followed by a post-exercise VOT 15 min after completing the exercise. The 15-min post-exercise time point was selected based on the window in which exercise-induced alterations in macrovascular function have been found (Doonan et al. 2013), and because it aligns with the other previous work examining post-exercise NIRS-based microvascular function (Caldwell et al. 2023; Perlet et al. 2024). Visit 2 was scheduled 1 week later at the same time of day as Visit 1. A pre-exercise VOT was repeated, followed by a submaximal exercise trial, blood volume measurements, and a post-exercise VOT, performed 15 min after completion of the exercise. For both visits, all participants arrived at the lab in the morning after a 12 h fast and abstained from alcohol, caffeine, and medications. Participants abstained from moderate or vigorous exercise for 24 h before each visit. Detailed descriptions of study methods are provided below.

### Anthropometrics and body composition

Heart rate (HR) measurements and brachial blood pressure (BP) were measured on the participant’s dominant arm with an automated device (SphygmoCor XCEL, AtCor Medical Sydney, NSW, Australia). Height (stadiometer, SECA North America, Chino, CA, USA) and weight (digital scale, Tanita Corporation, Arlington Heights, IL, USA) were measured, and BMI was calculated as body weight in kg/body height in m^2^. Body composition was measured using dual-energy X-ray absorptiometry (DXA) scan (Lunar Prodigy Z-ray Bone Densitometer, GE Healthcare, Chicago, IL, USA) as done previously (Zabriskie et al. 2020; Dobrosielski et al. 2021; Landers-Ramos et al. 2022). Participants were positioned on the DXA table by trained members of the research team. Quality assurance measures were performed daily (average CV over the study period 3.2%). All scans were analyzed using encore software (version 14.0) according to the manufacturer. Total body fat mass, lean mass, and bone mass were measured. Percent body fat was recorded for all participants.

### Participant set-up and familiarization

During the first visit, a mark was placed on the belly of the VL muscle along the distal length of the muscle to ensure enough space for the blood pressure cuff placement. Participants were instructed to keep this mark visible until Visit 2 to ensure consistency in NIRS placement between visits. A high-resolution ultrasound (GE Logiq, GE Healthcare Products, Chicago, IL, USA) equipped with a 7.5–12 MHz linear array transducer was used to determine adipose tissue thickness (ATT) and muscle thickness (MT) below the marked area. The area was then cleaned, and the NIRS device (Portamon, Artinis Medical Systems, Elst. The Netherlands) was secured over the marked location on the VL muscle using opaque athletic foam wrap to prevent ambient light from interfering with the NIRS signal. Subjects were then asked to lay supine on a table with their feet and knees supported with padding, such that their lower leg and feet were nearly parallel with the table. A tapered 4.5″ × 34″ blood pressure cuff (Delfi Medical Innovations, Vancouver, BC) was placed proximal to the knee on the upper thigh. The placement of the cuff was marked on the thigh with a marker, and the cuff tightness was indicated using a clip. These marks were placed to ensure that the cuff was placed in the same location and with the same tightness for the post-exercise assessment as well as for the assessments on Visit 2. The cuff was connected to a rapid inflation system (Hokanson E2, Hokanson, Inc., Carmel, IN, USA) and high-capacity air source (AG101, Hokanson, Inc., Carmel, IN, USA), which allows for inflation of the cuff in ~ 1 s to 300 mmHg, the pressure required for temporary arterial occlusion. To aid in participant comfort and to reduce movement due to initial responses to unfamiliar stimuli, participants were familiarized with rapid cuff inflation on the leg at 300 mmHg of pressure prior to testing.

### Blood volume

Total hemoglobin (tHb) was used to estimate intramuscular blood volume pre-exercise, at the end of each exercise trial, and 15-min post-exercise prior to initiating the VOT (Karasuno et al. 2005; Perlet et al. 2024). NIRS data were collected continuously throughout each study visit. Following 2 min of supine rest, tHb signals were averaged for 30 s before the pre-exercise VOT, during the final 30 s of each exercise trial, and for 30 s before each post-exercise VOT. NIRS-derived data were exported as 10 Hz files, and analyses were performed with a custom Python code using the tHb signal. Results were reported in optical density (O.D.).

### Vascular occlusion test

**Fig. 1:**
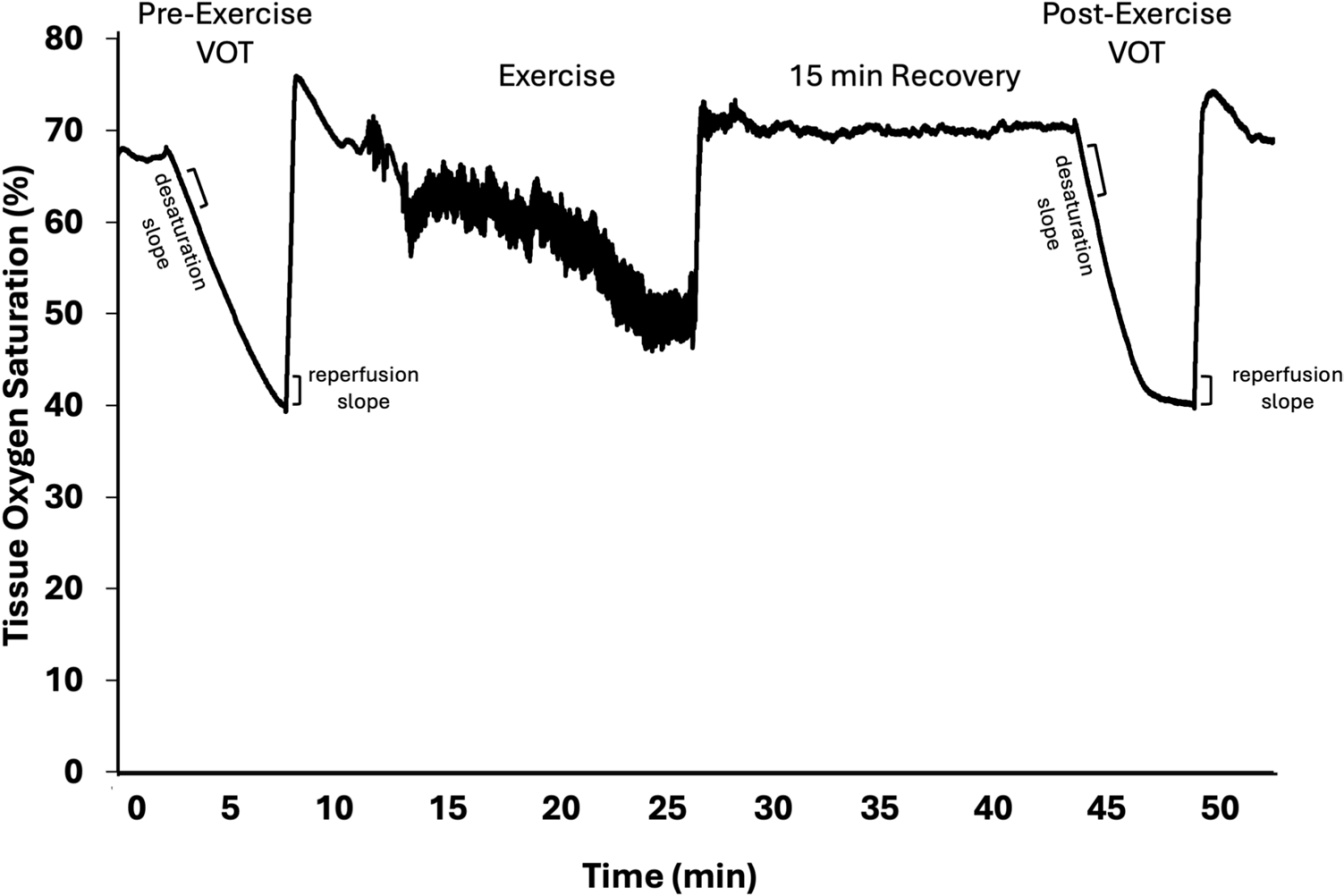
Representative tracings of the StO_2_ signal during vascular occlusion tests (VOT). Each trial consisted of a pre-exercise VOT, including 2 min of baseline testing, 5 min of limb occlusion, and 3 min of reperfusion. Participants then performed either maximal or submaximal cycling. A post-exercise VOT was then performed 15 min after exercise

Microvascular reactivity was assessed following 2 min of supine rest using an NIRS-based VOT before and 15 min following each exercise trial. Figure 1 depicts a representative tracing of the VOT pre- and post-exercise. Participants were asked to remain still, and NIRS data were collected continuously throughout the test. The VOT consisted of 2 min of baseline data followed by 5 min of arterial occlusion at 300 mmHg. The cuff was then rapidly released, and data in response to reactive hyperemia were collected for 3 min, allowing for the NIRS signal to reach the maximal level and begin to return to baseline. NIRS-derived data were exported as 10 Hz files, and analyses were performed with a custom Python code using the tissue oxygen saturation StO_2_ signal (calculated as [O_2_Hb/(O_2_Hb + HHb)]).

StO_2_ was used for all analyses during the VOT as previously reported (McLay et al. 2016a; Caldwell et al. 2023; Landers-Ramos et al. 2024b). Baseline StO_2_ (%) was calculated as the average StO_2_ for 1 min before the start of occlusion. StO_2_ minimum (min; %) was defined as the lowest value recorded during occlusion, and the StO_2_ maximum (max; %) was defined as the highest value detected after cuff release. The min and max signals were used to determine the physiological range of the StO_2_ signal which is reported as O.D. units. The desaturation slope, representing muscle oxygen consumption (mVO_2_), was defined as the steepest 30 s segment during the 5-min occlusion (% s^−1^) (Perlet et al. 2024). The reperfusion slope, representing microvascular reactivity, was defined as the 10 s upslope immediately after cuff release (% s^−1^) (McLay et al. 2016a, b; Iannetta et al. 2019; Landers-Ramos et al. 2024b; Perlet et al. 2024). Reproducibility tests for reperfusion slopes performed on *N* = 15 individuals produced a coefficient of variation (CV) of 6.1% over two visits performed 1 week apart (1.05 ± 0.52%s^−1^ vs. 1.06 ± 0.61%s^−1^; *p* = 0.959; unpublished data). Previous studies utilizing the same methods have reported intraday CV for reperfusion slopes between 9 and 11% in younger healthy individuals when tests were performed 30 min apart (McLay et al. 2016a, b).

### Maximal cycling trial

Following baseline testing on Visit 1, participants completed an incremental maximal exercise test to exhaustion on a cycle ergometer (Lode Ergometry, The Netherlands), and VO_2_ was measured continuously via indirect calorimetry (Parvo Medics TrueOne 2400, Salt Lake City, UT). After a 5-min warm-up, subjects began cycling at an intensity between 100 and 150 W, and the work rate increased by 25 W every 2 min until participants could not maintain a cadence of ≥ 50 revolutions per minute. HR was collected continuously using a chest strap HR monitor (Polar Electro Inc., Lake Success, NY). Peak HR, VO_2_peak, and caloric expenditure were recorded. Blood lactate was acquired at baseline and within 1 min of exercise completion using a handheld blood lactate analyzer (Lactate Plus, Nova Biomedical, Waltham, MA) as further confirmation of exercise intensity. Specifically, a blood lactate level of > 8 mmol was used to confirm maximal intensity (Howley et al. 1995).

### Submaximal cycling trial

The submaximal cycling test was performed at 60% of each participant’s VO_2_ peak. Participants were instructed to maintain their usual activity patterns for the week between trials. The submaximal exercise duration was individualized, as done previously (Jenkins et al. 2011), so participants met the same energy expenditure obtained through the maximal test (Liguori et al. 2021). Participants were instructed to maintain a cadence ≥ 60 rpm. VO_2_ and HR were measured continuously to confirm caloric expenditure and intended intensity. Blood lactate was acquired at baseline and within 1 min of exercise completion as further confirmation of exercise intensity.

### Statistical analysis

Sample size calculations were performed to determine the number of subjects needed to detect significant trial*time interaction in the major outcomes of interest (mVO_2_, microvascular reactivity, and blood volume). Calculations were based on effect size estimates from findings published in the literature regarding our primary study outcome, StO_2_ desaturation slope (Zhang et al. 2020; Perlet et al. 2024), StO_2_ microvascular reperfusion slope (Caldwell et al. 2023; Huang et al. 2024; Perlet et al. 2024), and tHb blood volume (Perlet et al. 2024). These calculations estimated a sample size of *N* = 11 for muscle oxygen desaturation (mVO_2_), *N* = 16 for microvascular reactivity, and *N* = 12 for blood volume to yield between 80 and 95% power to detect a significant interaction.

All statistical analyses were performed using SPSS version 25 (SPSS, Inc. Chicago, IL). Assumptions of normality and sphericity were verified for all outcome measures. Descriptive statistics were performed for all subject characteristics. A 2 (trial–max vs. submax) × 2 (time–pre vs. post-exercise) repeated-measures analyses of variance (ANOVA) was used for desaturation slope and microvascular reperfusion slope measures to determine differences before and after exercise and between study visits. A 2 (trial–max vs. submax) × 3 (pre vs. end vs. post-exercise) ANOVA was used to assess changes in blood volume. When an interaction or main effect was noted, post hoc pairwise comparisons were performed using Bonferroni corrections. The *α* level was set a priori for all statistical procedures at *α* = 0.05. Effect sizes were calculated for all statistically significant comparisons. Effect sizes for two-way ANOVAs are presented as partial eta-squared (*η*^2^_*p*_). The effect was determined to be trivial if < 0.01, small if the effect size was between 0.01 and 0.06, medium if between 0.06 and 0.14, and large if > 0.14 (Murphy and Myors 2004). For effect size calculations between different time points, Cohen’s *d* was used. For these results, the effect was determined trivial if the effect size was < 0.2, small if between 0.2 and 0.5, medium if between 0.5 and 0.8, and large if > 0.8 (Cohen 1988). Statistical significance was accepted at a *p* value of ≤ 0.05. Data are presented as means ± SD.

## Results

### Participant characteristics

A total of *N* = 22 individuals completed the study, but data from *N* = 4 were excluded due to participant movement (*N* = 3) or shifting of the NIRS device during exercise (*N* = 1) that prevented confident analyses of the NIRS signals. Participant characteristics from *N* = 18 included in the study can be found in Table 1. Participants averaged 22 ± 4 years of age and were generally healthy. A total of 13 males and 5 females completed this study. Of the female participants, during Visit 1, *N* = 3 and *N* = 2 reported menstrual cycles in the luteal and follicular phases, respectively. During Visit 2, *N* = 2 and *N* = 3 females reported menstrual cycles in the luteal and follicular phases, respectively. Due to evidence indicating that the menstrual cycle has small-to-no effects on microvascular function (Williams et al. 2020; D’Souza et al. 2023), this was not controlled for. Participants were recreationally active, which is reflected in the self-reported physical activity and VO_2_peak results (Liguori et al. 2021).

**Table 1:** Participant characteristics (*N* = 18)

M/F (*N*)	13/5
Age (years)	22 ± 4
Resting HR (bpm)	59 ± 9
Resting SBP (mmHg)	126 ± 10
Resting DBP (mmHg)	70 ± 8
BMI (kg/m^2^)	24 ± 2
Body fat (%)	23 ± 8
Absolute VO_2_peak (L/min)	2.9 ± 0.8
Relative VO_2_peak (ml/kg/min)	40.8 ± 8.6
Max HR (bpm)	192 ± 7
MT (mm)	25.8 ± 3.8
ATT (mm)	5.9 ± 2.5
Aerobic training (min/wk)	228 ± 163
Resistance training (min/wk)	205 ± 158

Means ± SD

*bpm* beats per minute, *SBP* systolic blood pressure, *DBP* diastolic blood pressure, *HR* heart rate, *BMI* body mass index, *VO*_*2*_ volume of oxygen, *MT* muscle thickness, *ATT* adipose tissue thickness

### Confirmation of exercise protocols

The average VO_2_ during the submax trial was 26.7 ml/kg/min, equating to 64% of VO_2_peak. As intended, caloric expenditure was matched between the max trial (97 ± 3 kcal) and submax trial (101 ± 44 kcal; *p* = 0.329), with the submax trial taking significantly longer to complete (521 ± 124 s vs. 644 ± 126 s; *p* < 0.001). As expected, there was a significant trial*time interaction for blood lactate (*p* < 0.001). Blood lactate was significantly elevated after both the max trial (2.3 ± 1.1 mmol/L vs. 10.4 ± 2.3 mmol/L pre vs. post, respectively; *p* < 0.001) and the submax trial (1.9 ± 0.7 mmol/L vs. 4.9 ± 2.7 mmol/L pre vs. post, respectively; *p* < 0.001) with post-exercise blood lactate being significantly higher after the max trial compared with the submax trial (*p* < 0.001).

### Blood volume

**Fig. 2:**
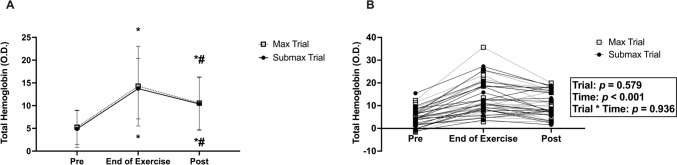
Blood volume assessed using total hemoglobin (tHb) pre-exercise, during the last 30 s of exercise, and 15-min post-exercise for both maximal and submaximal cycling trials. **A** depicts means, and **B** depicts individual data. Compared with the pre-exercise, blood volume was elevated at the end of exercise (*p* = 0.001) regardless of trial. In both the max and submax trials, despite being reduced from the end of exercise and 15 min post-exercise (*p* < 0.001), blood volume remained elevated 15 min post-exercise compared with pre-exercise levels (*p* < 0.001). *Indicates significantly different than pre-exercise timepoint within trial; ^#^indicates significantly different than the end of exercise timepoint within trial

There was no significant trial*time interaction for tHb (*p* = 0.936; *η*_*p*_^2^ = 0.004; Fig. 2), nor was there a significant main effect of trial (*p* = 0.579; *η*_*p*_^2^ = 0.018). There was a significant main effect of time (*p* < 0.001; *η*_*p*_^2^ = 0.751). Compared with pre-exercise levels, tHb was elevated within the last 30 s of exercise (*p* < 0.001; *d* = 1.423) regardless of trial. While tHb decreased between the end of exercise and the 15-min post-exercise time point (*p* = 0.001; *d* = 0.469), it remained elevated at the 15 min post-exercise time point compared with pre-exercise levels (*p* < 0.001; *d* = 1.147) in both the max and submax trial.

### Vascular occlusion test parameters

Results from the vascular occlusion test can be found in Table 2. There was no trial*time interaction noted for baseline StO_2_ (*p* = 0.532, *η*_*p*_^2^ = 0.023), nor was there a main effect of trial (*p* = 0.106, *η*_*p*_^2^ = 0.146). Regardless of trial, baseline StO_2_ was significantly elevated after each exercise bout (*p* = 0.011, *η*_*p*_^2^ = 0.326). Similarly, there was no interaction (*p* = 0.430, *η*_*p*_^2^ = 0.037) or main effect of trial (*p* = 0.790, *η*_*p*_^2^ = 0.004) for StO_2_ min. However, there was a significant main effect of time for StO_2_ min (*p* < 0.001, *η*_*p*_^2^ = 0.527) with StO_2_ reaching lower values at pre-exercise timepoints on both the max and submax trials. StO_2_ max did not exhibit any trial*time interaction (*p* = 0.415, *η*_*p*_^2^ = 0.039), main effect of trial (*p* = 0.411, *η*_*p*_^2^ = 0.040), or main effect of time (*p* = 0.600, *η*_*p*_^2^ = 0.016). StO_2_ range appeared to be influenced by the StO_2_ min, which mirrors those findings including no trial*time interaction (*p* = 0.137, *η*_*p*_^2^ = 0.125) and no main effect of trial (*p* = 0.109, *η*_*p*_^2^ = 0.144), but a significant main effect of time (*p* < 0.001, *η*_*p*_^2^ = 0.624) with a larger StO_2_ range at the pre-exercise time points, regardless of trial.

**Table 2:** Vascular occlusion test parameters

	Max trial		Submax trial		*p* value (partial eta-squared)		
	Pre	Post	Pre	Post	Trial	Time	Trial*time interaction
Baseline StO_2_ (%)	77 ± 6	78 ± 6	75 ± 5	76 ± 4	0.106 (0.146)	**0.011** (0.326)	0.532 (0.023)
StO_2_ min (%)	41 ± 12	44 ± 15	42 ± 11	44 ± 12	0.790 (0.004)	** < 0.001** (0.527)	0.430 (0.037)
StO_2_ max (%)	80 ± 5	79 ± 6	79 ± 4	79 ± 3	0.411 (0.040)	0.600 (0.016)	0.415 (0.039)
StO_2_ range (O.D.)	39 ± 12	35 ± 12	37 ± 11	35 ± 11	0.109 (0.144)	** < 0.001** (0.624)	0.137 (0.125)

Data are presented as means ± SD

*StO*_*2*_ tissue oxygen saturation, *O.D.* optical density

**Fig. 3:**
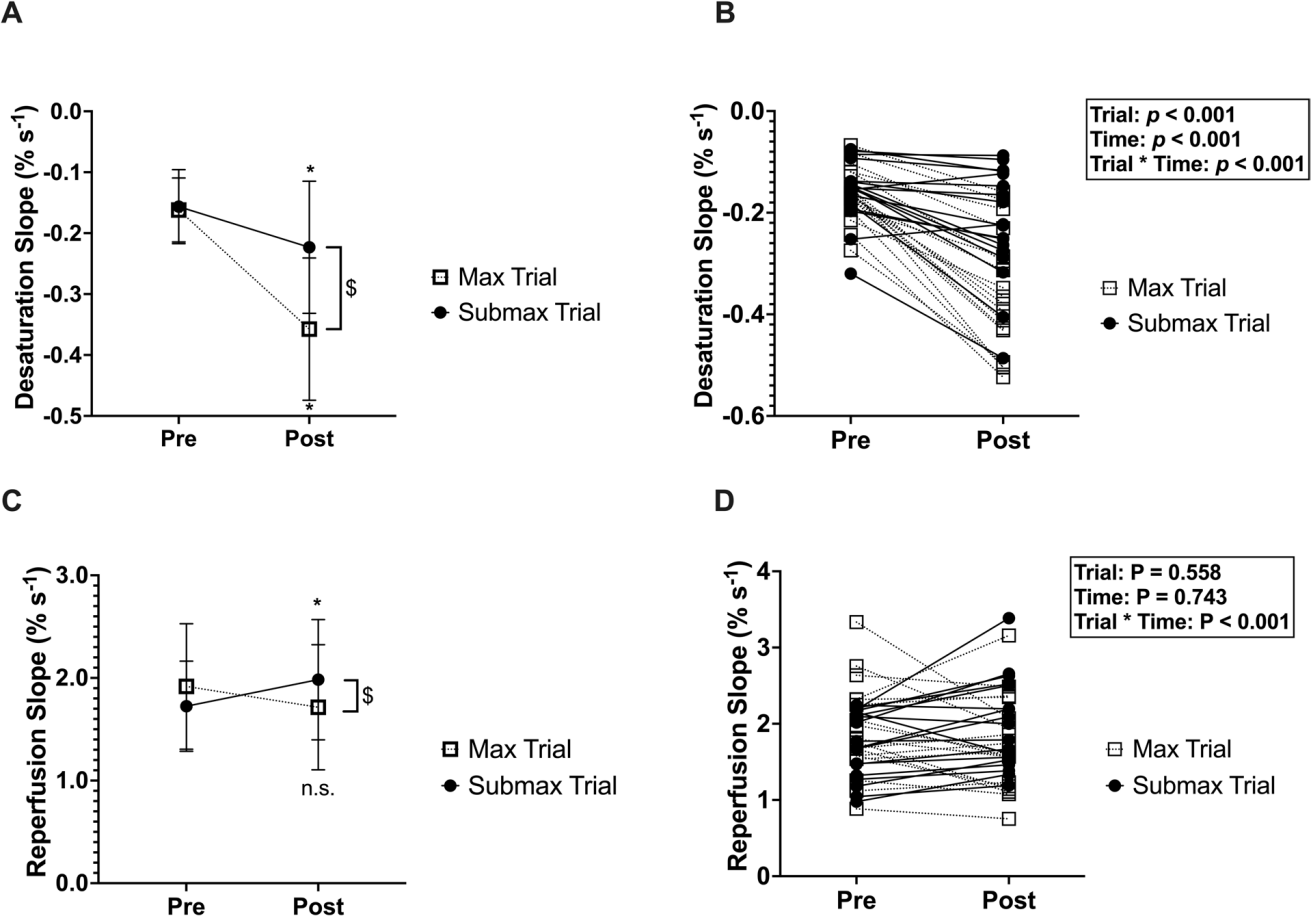
**A** (means) and **B** (individual data) depicting desaturation slope (%s^−1^) representing mVO_2_. There were no differences between trials at the pre-exercise timepoint (*p* = 0.485). Compared with pre-exercise, both the max (*p* < 0.001) and submax (*p* = 0.001) exercise resulted in greater mVO_2_. Post-exercise, mVO_2_ was significantly greater in the max trial vs. submax (*p* < 0.001). Panels C (means) and D (individual data) depicting reperfusion slope (%s^−1^) representing skeletal muscle microvascular reactivity. There were no pre-exercise differences between trials (*p* = 0.072). Compared with pre-exercise, there were no significant changes in microvascular reactivity following the max trial (*p* = 0.079), but microvascular reactivity was significantly greater following the submax trial (*p* = 0.007). Post-exercise microvascular reactivity was significantly higher after the submax trial compared with the max trial (*p* < 0.001). *Indicates significantly different than pre-exercise timepoint within trial; ^$^indicates significant difference between trials at timepoint

There was a significant trial*time interaction (*p* < 0.001; *η*_*p*_^2^ = 0.704), as well as a significant main effect of trial (*p* < 0.001; *η*_*p*_^2^ = 0.807) and main effect of time (*p* < 0.001; *η*_*p*_^2^ = 0.599) for the desaturation slopes (i.e., mVO_2_, Fig. 3a). There were no significant differences between trials at the pre-exercise timepoint (*p* = 0.485, *d* = 0.106). Both the max (*p* < 0.001, *d* = 2.142) and submax (*p* = 0.001, *d* = 0.764) exercise resulted in significantly greater desaturation but was significantly greater in the max trial (*p* < 0.001, *d* = 1.181). There was a significant trial*time interaction for the reperfusion slope (i.e., microvascular reactivity; *p* < 0.001; *η*_*p*_^2^ = 0.535; Fig. 3b). Compared with pre-exercise, there were no significant changes in reperfusion slopes following the max trial (*p* = 0.079, *d* = − 0.344), but reperfusion slopes were significantly greater following the submax trial (*p* = 0.007, *d* = 0.499). There were no pre-exercise differences between trials (*p* = 0.072, *d* = 0.376), and post-exercise reperfusion slopes were significantly higher after the submax trial compared with the max trial (*p* < 0.001, *d* = 0.449).

## Discussion

Microvascular reactivity responses contribute to intramuscular blood volume and allow the body to meet the metabolic recovery needs following a bout of exercise. The main findings of this study are (1) blood volume, measured through tHb, was significantly elevated in the last 30 s of both maximal and submaximal exercise trials. Blood volume remained elevated above pre-exercise levels 15-min post-exercise with no differences between max and submax trials, and (2) compared with pre-exercise, microvascular reactivity was greater following submax exercise but was unchanged following maximal exercise. These findings suggest that cycling exercise performed at a submaximal, but not maximal, intensity can increase post-exercise microvascular reactivity, while blood volume alterations in the VL occur similarly with exercise regardless of intensity.

In line with previous studies assessing intramuscular blood flow (Andersen and Saltin 1985; Bangsbo and Hellsten 1998; Tschakovsky et al. 2002; Sarelius and Pohl 2010; Joyner and Casey 2015), we found that blood volume was elevated during exercise compared to pre-exercise levels and remained elevated for at least 15 min post-exercise. However, despite studies suggesting that elevations in intramuscular blood flow are proportionate to the exercise intensity (Tschakovsky et al. 2002; Joyner and Casey 2015), we did not observe differences in blood volume between max and submax trials at any time point. These findings occurred despite seeing intensity-dependent differences in mVO_2_ following exercise, indicating that intramuscular blood volume is not solely regulated by the need for oxygen in exercised muscle (Bangsbo and Hellsten 1998; Heinonen et al. 2015; Izumi et al. 2024). Our findings may be explained by the use of tHb to assess intramuscular blood volume, as opposed to blood flow. While blood volume during dynamic exercise is due, at least in part, to changes in blood flow, these outcomes are not directly comparable. Still, our findings may also be explained by our subject population exhibiting adequate oxygen delivery to the VL even at maximal intensities of exercise. Of note, studies demonstrating the increase in intramuscular blood flow or blood volume with higher exercise intensities have typically been done in conditions where the active muscle mass is small (Andersen and Saltin 1985; Newman et al. 1997; Rådegran and Saltin 1999; Izumi et al. 2024). In contrast, our study used aerobic cycling exercise with greater total muscle recruitment and, presumably, greater blood flow redistribution throughout a greater portion of all recruited muscles. Likewise, a maximal cycling test does not indicate maximal muscle recruitment of the VL. Our results suggest that exercise-intensity differences in intramuscular blood volume of the measured part of the VL are not evident as cycling exercise would lead to greater distribution of blood volume to all working muscles. Nevertheless, findings similar to ours have been previously reported using both isolated contractions (Izumi et al. 2024) and cycling exercises (Habazettl et al. 2010). For example, Izumi et al. reported that intramuscular blood flow in the VL plateaued above moderate-intensity incremental isometric knee extension exercise. At the same time, muscle oxygenation continued to decrease at higher intensities (Izumi et al. 2024). Similarly, Habazettl et al. found that blood flow, using NIRS with indocyanine green tracer, in the quadriceps muscle increased up to 60% of maximal work rate when performing incremental cycling exercise, and then plateaued at higher intensities (Habazettl et al. 2010). Importantly, as our study was conducted in healthy, young individuals, future studies should assess whether this disconnect between post-exercise muscle blood volume and mVO_2_ is present in older or clinical populations with poorer oxygen-carrying capacity or blood flow impairment.

In this study, we found intensity-dependent differences in microvascular reactivity after exercise. Specifically, compared with baseline, microvascular reactivity was significantly increased following submaximal exercise but was unchanged following maximal exercise. During exercise and in the acute post-exercise timeframe, agents, such as nitric oxide (NO), prostaglandins, and adenosine, serve as vasodilation signals and/or inhibitors of vasoconstriction to allow for the delivery of oxygen and nutrients to the exercising and recovering muscle (Rådegran and Saltin 1999; Boushel et al. 2002; Mortensen and Saltin 2014; Joyner and Casey 2015; Robinson et al. 2018). On a macrovascular level, there is evidence of impaired vascular function (i.e., FMD) up to ~ 60 min following maximal exercise (Dawson et al. 2013). One of the mechanisms proposed to explain the reduction in FMD following high-intensity exercise is oxidative stress (Goto et al. 2003; Robinson et al. 2018). Excess free radicals produced during high-intensity exercise can blunt NO production and the associated vasodilatory responses (Goto et al. 2003; Robinson et al. 2018). Indeed, Goto et al. found that while moderate-intensity exercise training augments endothelium-dependent vasodilation through greater NO production, high-intensity exercise increases markers of oxidative stress (Goto et al. 2003). Assuming similar mechanisms influence microvascular reactivity, greater oxidative stress resulting from maximal intensity exercise may inhibit NO production and thus attenuate microvascular reactivity. Additionally, while not directly assessed in this study, the maximal exercise is associated with a greater sympathetic vasoconstriction response, which would theoretically impair microvascular reactivity (Rowell 1997; Joyner and Casey 2015). Still, there is evidence that this response is blunted in the exercising muscle bed in an intensity-dependent fashion (Tschakovsky et al. 2002). Contrary to our hypothesis, we observed no significant difference in microvascular reactivity following the maximal exercise compared with pre-exercise. While the effect size for this outcome was small, the numerical reduction observed is aligned with the reduced macrovascular function observed following maximal exercise and may be reflective of the above-mentioned mechanisms. Some studies have suggested that macro- (Harris et al. 2008; Dawson et al. 2013; Kapilevich et al. 2020) and microvascular (Robinson et al. 2018; Rasica et al. 2022) declines following high-intensity exercise are not evident in individuals with higher cardiorespiratory fitness. The VO_2_ peak of our subject population indicated moderate cardiorespiratory fitness for individuals of this age (Liguori et al. 2021). We have previously reported augmented FMD following moderate-intensity exercise in moderately fit individuals (Landers-Ramos et al. 2024a), which agrees with our microvascular findings in the present study. However, further studies are needed to evaluate the relationship between fitness levels and microvascular reactivity following maximal cycling exercise. Studies using other methods to assess microvascular function post-exercise using smaller muscle mass have generally reported reduced (Nardone et al. 2021; Sweet et al. 2024) or unchanged (Rytter et al. 2020) microvascular function in response to high-intensity exercise, while some studies indicate greater microvascular function following moderate-intensity exercise (Sweet et al. 2024). As noted above, despite these intensity-dependent differences in post-exercise microvascular reactivity, we found that blood volume responded similarly at both maximal and submaximal intensities. This implies that the mechanisms involved in post-exercise blood volume recovery are a consequence of a myriad of possibilities, whereas our VOT protocol assessing microvascular reactivity may be interrogating only one of these mechanistic pathways. While more research is needed to fully understand these responses, carefully monitoring exercise intensity over regular training sessions may have significant microvascular impacts on recovery in sport and rehabilitation settings.

The acute post-exercise time point serves as a window to understanding the prolonged effects of exercise that may inform adaptations or recovery needs (Luttrell and Halliwill 2015). To our knowledge, a few published studies have examined microvascular reactivity in the acute post-exercise timeframe, particularly following dynamic exercise using large muscle mass. Perlet et al. recently found that skeletal muscle microvascular reactivity of the VL (assessed using NIRS) was significantly elevated 15 min following an acute bout of resistance training with blood flow restriction (BFR) but not traditional resistance training (Perlet et al. 2024). In the same study, the authors also found that tHb increased in the VL during exercise in the BFR condition but not with traditional resistance exercise. They speculated that the higher tHb was due to the accumulation of blood in the vascular and extracellular tissue that promoted a rise in local hydrostatic pressure and the resulting increase in microvascular vasodilation observed in the BFR condition. Comparing these findings with the present study, while cycling increases mVO_2_ of the muscle tissue, cycling (as opposed to BFR) may not promote a significant enough local accumulation of blood into the VL tissue underlying the NIRS probe for this mechanism to explain the greater microvascular reactivity found in our submaximal cycling condition. Findings from another study using NIRS to assess microvascular reactivity post-exercise, at first glance, appear to contrast with ours (Caldwell et al. 2023). Caldwell et al. demonstrated a reduction in microvascular reactivity of the medial gastroc muscle 15 min after completing a bout of treadmill running at 65% VO_2_peak, an intensity similar to our submaximal condition (Caldwell et al. 2023). However, 30 min before the submax test, participants performed an incremental running test to exhaustion, whereas our maximal trial was performed 1 week prior. While the timeframe for post-exercise microvascular alterations is not well understood, macrovascular function is often not recovered until ~ 60 min following high-intensity exercise (Dawson et al. 2013). Thus, the reduced microvascular reactivity following the submax test in the Caldwell et al.’s study may represent residual or compounded effects of the prior maximal bout.

This study is strengthened by the repeated-measures design, which helps to control for interindividual differences in skeletal muscle vascularization, adipose tissue thickness, and skin characteristics that may be picked up by NIRS (Heinonen et al. 2015). Another strength of this study is the inclusion of male and female participants representing the fitness category of most young active individuals, making findings generalizable to a large population. Still, the study does have limitations that warrant mention. First, NIRS only assesses one portion of VL, which may not be representative of the entire muscle or across other quadriceps muscles. Second, in this study, we assessed blood volume using tHb, which is a static measure. Intramuscular blood volume changes in response to dynamic exercise are likely related to commensurate changes in blood flow in healthy individuals. However, we did not measure blood flow directly, which limits direct comparisons to other studies. Another limitation of this study is the lack of a non-exercise control limb to determine whether the observed responses are systemic or localized to the exercising muscle. Finally, while the timeframe of the macrovascular responses to acute exercise is well explored (Dawson et al. 2013), microvascular responses are less understood. The 15 min post-exercise time point was selected as it fell in the range of time in which macrovascular function exhibits intensity-dependent impairments. However, microvascular responses may not fall within the same timeframe, and earlier or later assessments might reveal different findings.

While the present study looked at only healthy young adults, these findings serve as a springboard to understand post-exercise perfusion dynamics in those with chronic conditions who exhibit exercise intolerance. The microvascular response to exercise recovery has been recognized as a valuable contributor to the understanding of many clinical conditions typically associated with only macrovascular dysfunction (Krentz et al. 2009; Houben et al. 2017). Thus, future studies should examine the effect of exercise intensity on microvascular reactivity in clinical populations and older adults to advise best practices for exercise recovery in a rehabilitation setting. In conclusion, similar muscle blood volume changes were evident with exercise, regardless of intensity, despite a higher mVO_2_ following maximal exercise and greater microvascular reactivity following submaximal exercise. The observed increase in microvascular reactivity following submaximal exercise may have implications for training and recovery practices in sport.

## Data Availability

Raw data are available upon reasonable request.

## Abbreviations

ANOVA: Analysis of variance
ATT: Adipose tissue thickness
BP: Blood pressure
BFR: Blood flow restriction
BMI: Body mass index
FMD: Flow-mediated dilation
HHb: Deoxygenated hemoglobin
O_2_Hb: Oxygenated hemoglobin
HR: Heart rate
MT: Muscle thickness
mVO_2_: Muscle oxygen consumption
NIRS: Near-infrared spectroscopy
NO: Nitric oxide
O.D.: Optical density
SD: Standard deviation
StO_2_: Tissue oxygen saturation
tHb: Total hemoglobin
VL: Vastus lateralis
VO_2_: Volume of oxygen consumption
VOT: Vascular occlusion test
